# Gorham-Stout disease: remission with sirolimus therapy

**DOI:** 10.1259/bjrcr.20230032

**Published:** 2023-09-12

**Authors:** Stefanie W. Y. Yip, James F Griffith, Cina S. L. Tong, Maribel D Lacambra, Frankie W. T. Cheng

**Affiliations:** 1 Department of Imaging and Interventional Radiology, Prince of Wales Hospital, The Chinese University of Hong Kong, Hong Kong, China; 2 Department of Anatomical and Cellular Pathology, Prince of Wales Hospital, The Chinese University of Hong Kong, Hong Kong, China; 3 Hong Kong Children’s Hospital, Kowloon Bay, Hong Kong, China

## Abstract

Gorham-Stout disease (GSD) is a rare, non-hereditary, bone disease characterised by progressive osteolysis as a result of uncontrolled proliferation of endothelial-lined vessels replacing normal bone. We present a baby-girl with the classic radiological features of GSD and compatible clinical and histological findings, who developed progressive disease for over 2 years despite propranolol treatment. Propranolol treatment was stopped and sirolimus monotherapy started which resulted in near-complete resolution after 1 year, with no recurrence after discontinuation of treatment. This case not only illustrates the typical features of GSD on a variety of imaging modalities, but is also the first report showing stark contrast in response between propranolol and sirolimus treatment for GSD, highlighting how targeting lymphatic, rather than solely angiomatous, proliferation at the vascular endothelial growth factor-level may be a future direction.

## Clinical presentation

A previously healthy 14-month-old girl presented with painless non-traumatic swelling of the right distal calf and ankle. The overlying skin was normal. Developmental milestones were normal.

## Imaging findings

**Figure 1.**
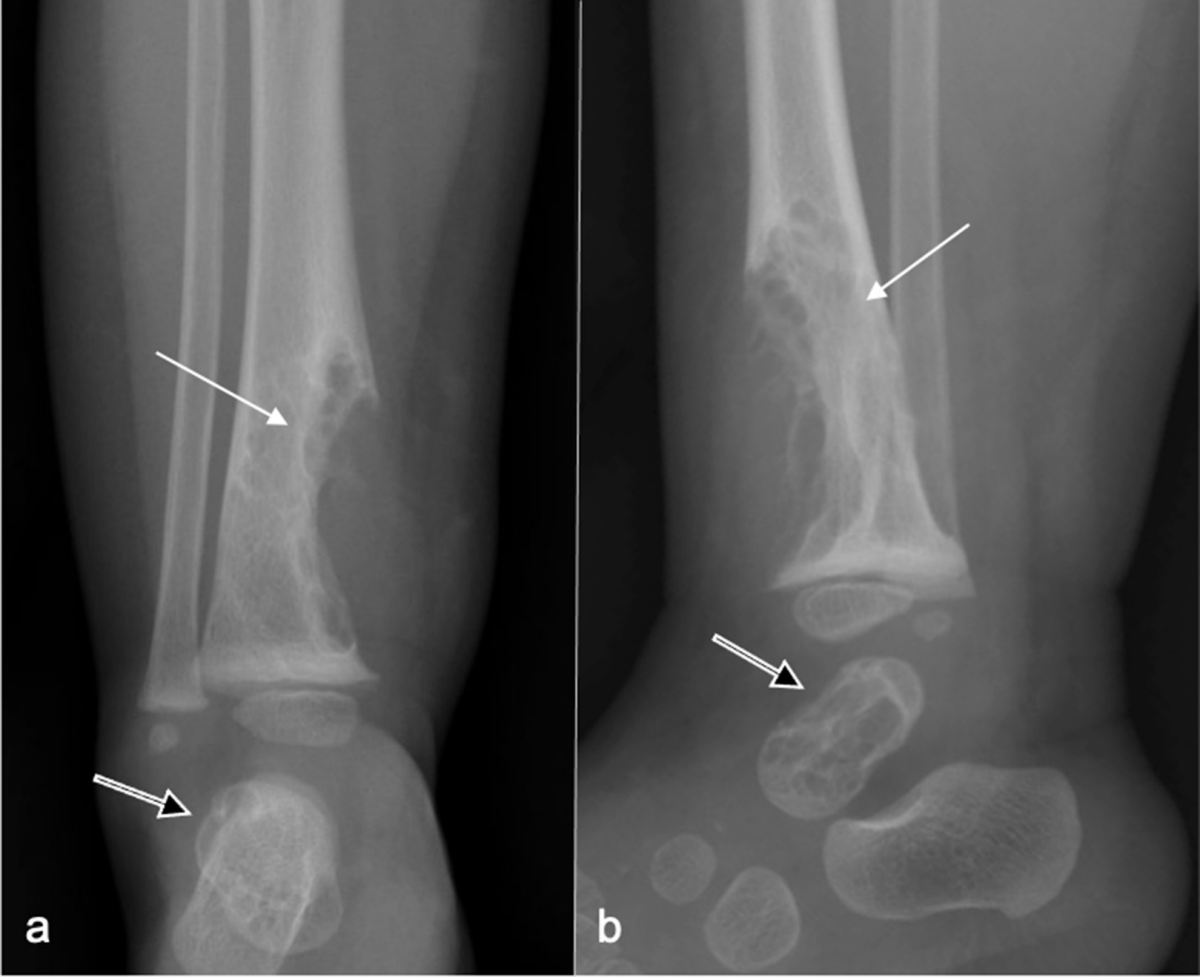
Frontal (**a**) and lateral (**b**) radiographs at presentation show a large well-defined, mildly spiculated, lobulated lytic lesion in the distal tibial metadiaphysis with a sclerotic rim (arrow). The overlying cortex is disrupted with a medium-sized extraosseous soft tissue swelling. No periosteal reaction is present. Neither the physis nor the epiphysis are affected. A similar lesion involves most of the talus (open arr).

**Figure 2.**
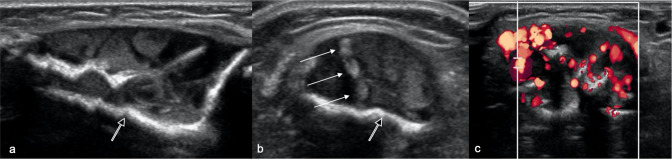
Longitudinal (**a**) and (**b, c**) transverse ultrasound images at presentation at the site of distal tibia cortical breach shows loss of the anterior cortex of the tibia with a well-defined, partially septated, intramedullary lesion, extending into the extraosseous soft tissues. The lesion comprises large cystic areas, containing rouleaux-like echogenic aggregates (arrows), and has a strongly echogenic rim (open arrow). (**c**) Power Doppler imaging shows moderate intralesional hyperaemia.

Radiographs (Figure 1) revealed large well-defined lytic areas in the distal tibial metadiaphysis and talus with thin sclerotic rims. The distal tibial lesion had breached the anteromedial cortex with extraosseous soft tissue extension. There was no matrix mineralisation, periosteal reaction, or pathological fracture. Ultrasound (Figure 2) at the site of anteromedial tibial cortical breach showed well-defined, medium-sized, partially septated cystic-like areas within the medullary canal of the distal tibia with an echogenic rim consistent with the sclerotic rim seen radiographically (Figure 2a). The cystic areas contained fine echogenic rouleaux-type aggregates (Figure 2b). Moderate slow-flow vascularity was evident on power Doppler imaging (Figure 2c). Following ultrasound examination, the most likely diagnosis was a vascular malformation with intra- and extraosseous components.

**Figure 3.**
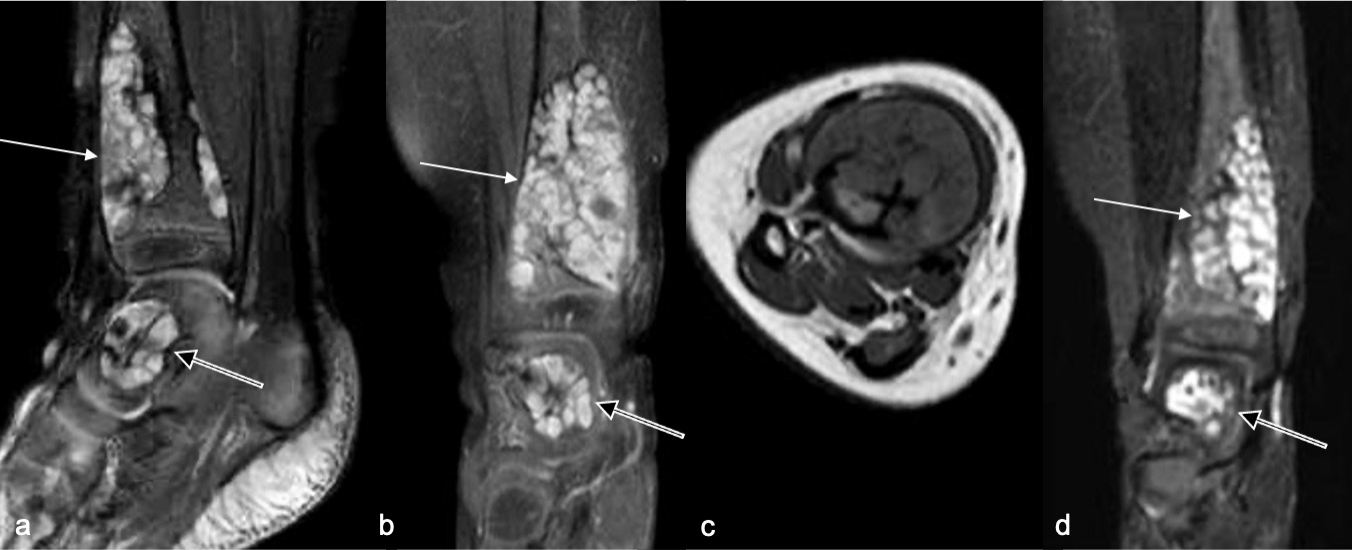
MRI of distal leg and hindfoot at presentation. (**a**) Sagittal, (**b**) coronal *T*_2_ weighted fat-suppressed images, (**c**) *T*_1_ weighted axial image and (**c**) coronal contrast-enhanced *T*_1_ weighted fat-saturated image showing well-defined multilobulated avidly enhancing lesions in the distal tibia (arrow) and talus (open arrow) corresponding to the lytic lesions seen radiographically (Figure 1). There are no flow voids. These MR appearances favoured an intraosseous intermediate-flow vascular malformation.

**Figure 4.**
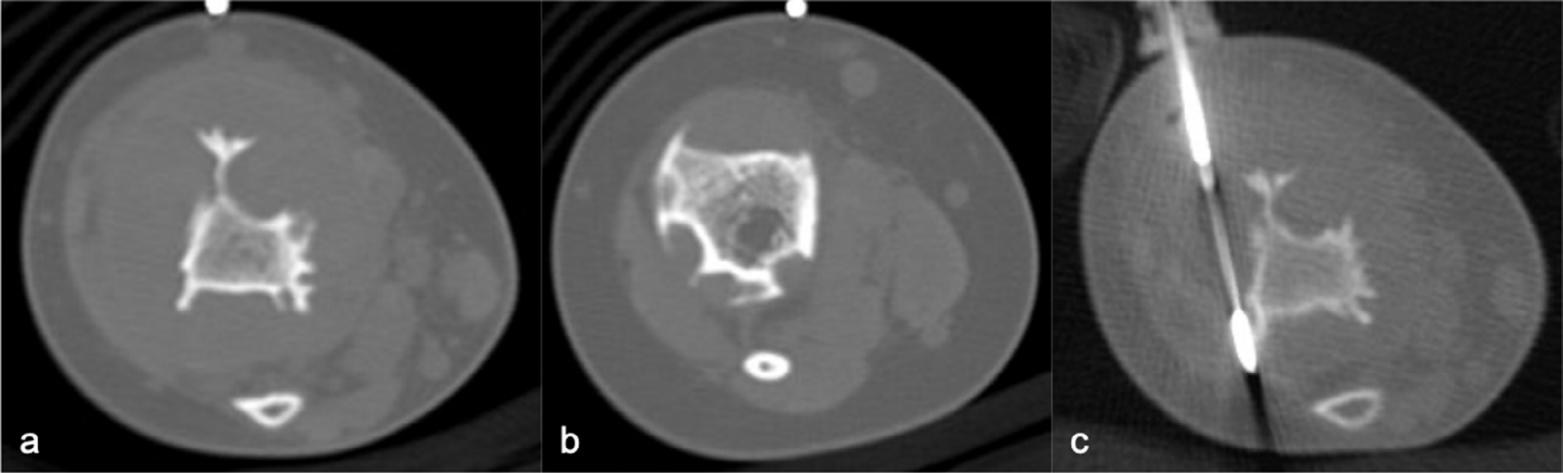
CT in axial bone window, showing multiple scalloped well-demarcated intramedullary lytic areas with sclerotic margins in distal tibia. Trucut biopsy of the soft tissue component was performed under CT guidance.

**Figure 5.**
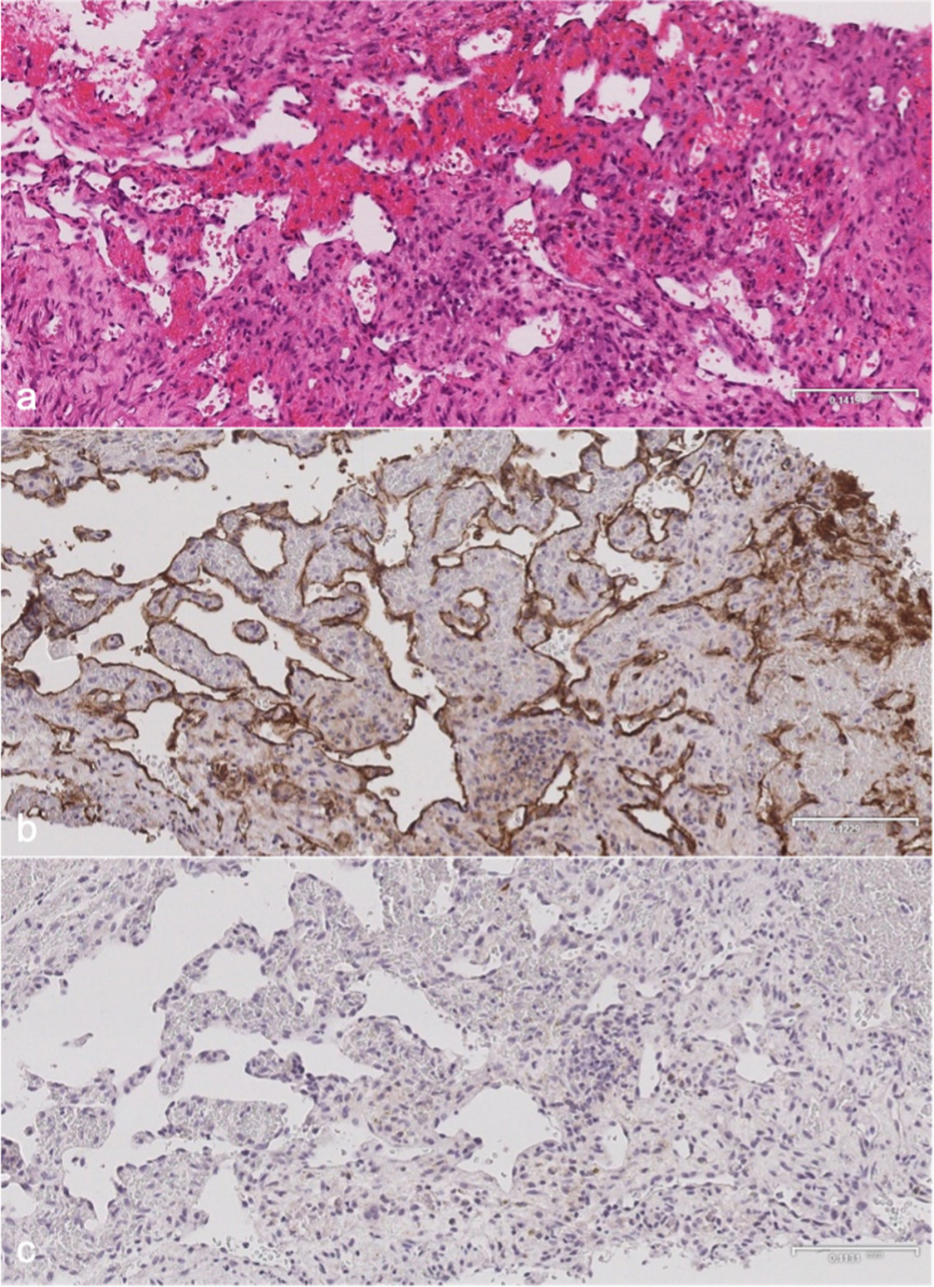
Microscopy of distal tibial biopsy. (**a**) H&E stain showing anastomosing blood vessels lined by endothelial cells with no significant nuclear atypia. There is no lymphatic component or bony matrix. (**b**) CD-31 immunohistochemical stain is positive confirming a vascular nature. (**c**) D2-40 immunohistochemical stain is negative indicating no demonstrable lymphatic tissue. H&E, Hematoxylin and eosin.

MRI (Figure 3) revealed small lobulated multiseptated avidly enhancing juxtacortical lesions within the radiographically apparent osteolytic areas with soft tissue extension. There were no flow voids, phleboliths, fluid–fluid levels or perilesional marrow oedema (Figure 3). On CT (Figure 4), the lesions were seen as multiple scalloped well-defined intramedullary lytic areas with sclerotic margins. CT-guided bone biopsy of the distal tibial lesion revealed endothelial-lined angiomatous spaces mixed with fibrous tissue and mild inflammatory infiltrate, with no apparent osteoid matrix, consistent with Gorham-Stout disease (Figure 5a). Immunohistochemical stains for CD-31 was positive and negative for D2-40, indicative of an angiomatous rather than lymphatic content lesion (Figure 5b and c). Subsequent imaging and laboratory work-up revealed no visceral or endocrine anomaly.

## Diagnosis and differential diagnosis

Progressive and predominant cortical-based bony resorption with tapering ends, involving contiguous bones, and limited osteoblastic response with no periosteal reaction, as seen in this case, are the typical radiographic appearances of Gorham-Stout disease.^1^ As the disease is uncommon and sporadic, the diagnosis is often not considered initially. Cystic angiomatosis and vascular tumours can have similar imaging and histological appearances. However, the presence of progressive cortical-based non-expansile osteolysis with more bone than soft tissue involvement, affecting the appendicular rather than the axial skeleton, involvement of contiguous bones across joints, absence of matrix calcification, and absence of cutaneous, lymphatic, or visceral abnormality favour Gorham-Stout disease.^2^ Vascular anomaly tumours typically feature medullary-based osteolysis, tend to be slightly more expansile, and often have thickened trabeculae and matrix calcifications.^3^ Ultrasound and MRI examinations were very helpful in showing well-demarcated, honeycomb-appearing, slow-flow vascular lesions replacing bone with extraosseous extension, helping to firm up the diagnosis and exclude entities such as osteofibrous or fibrous dysplasia and other solid tumours. The biopsy showing non-neoplastic vascular proliferation with fibrosis and mild inflammatory infiltrate was compatible. However, the diagnosis cannot be made on histology alone, as it is the radiological pattern of progressive bony dissolution that mainly distinguishes Gorham-Stout disease from other vascular anomalies.^1^

## Treatment, outcome and follow-up

**Figure 6.**
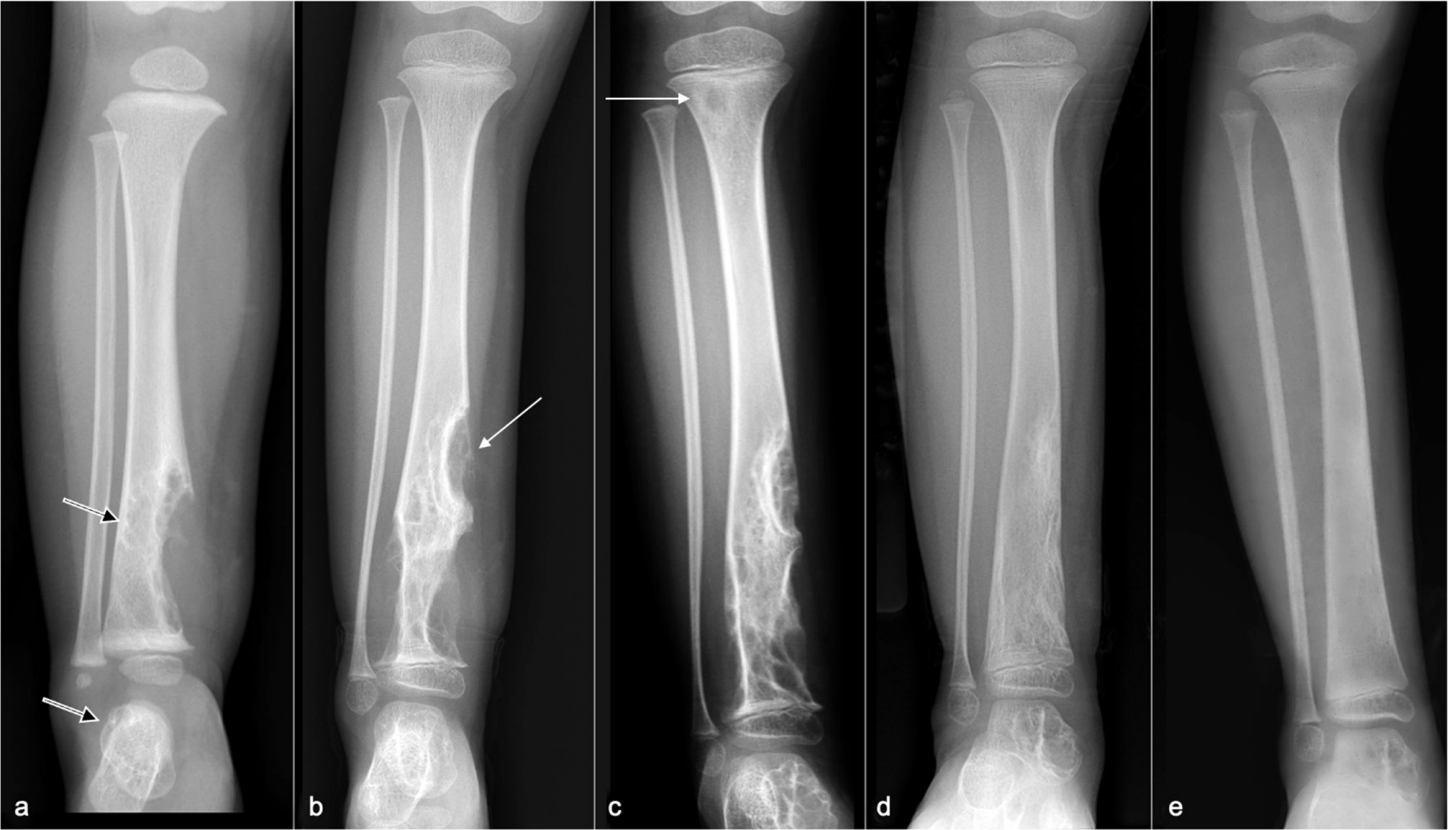
Serial radiographs at (**a**) presentation and (**b**) 1 year, (**c**) 2 years, (**d**) 2.5 years, and (**e**) 3 years later. (**a**) Initial radiograph shows a large osteolytic lesion in the distal tibia (arrow) and talus (open arrow). (**b**) 1 year after propranolol treatment, there is progressive osteolysis with proximal extension of the lytic area along the tibial diaphysis (arrow) and early involvement of the distal tibial epiphysis. (**c**) Despite escalation of propranolol treatment, there is further disease progression at 2.5 years with a new lytic subcortical lesion in the proximal tibial metaphysis (arrow) and enlargement of the lesions in the distal tibia and talus. (**d**) After discontinuing propanolol and 6 months of sirolimus monotherapy, there is substantial regression of the osteolysis. The proximal tibial metaphyseal lesion is no longer apparent. (**e**) After 1 year of sirolimus treatment, the distal tibial lesions have near-completely resolved with the tibial contour returning to normal and no soft tissue swelling. The talar lesion is smaller.

The patient was initially started on oral propranolol 2.5 mg three times daily. However, serial radiographs and MRI examinations showed steady disease progression (Figure 6) with enlargement and proximal extension of the distal tibial osteolysis and early involvement of the distal tibial epiphysis (Figure 6a). There was also mild interval lengthening of the affected tibia and fibula, which became approximately 1 cm longer than the non-affected side at 1-year from presentation. Propranolol dosage was increased to 7 mg three times daily. 1 year later, there was further disease progression and a new osteolytic lesion in the proximal tibial metaphysis (Figure 6c).

Due to disease progression, propranolol was discontinued and the patient was started on oral sirolimus, initially at 0.5 mg bd for first month, then gradually stepped up to 0.75 mg bd the second month, 1.0 mg bd the third month, and 1.5 mg bd from the fourth month onwards. 1 month after starting sirolimus, she started showing clinical response with reduced right leg swelling. Clinical condition, complete blood count, liver and renal function tests, and serum sirolimus trough level were checked every fortnight for first 2 months, then every month for the subsequent 4 months, with the aim of achieving a therapeutic sirolimus level of 10–15 ng ml^−1^. The patient tolerated sirolimus well, with no adverse symptoms encountered and remains fully active with no limitations in weight-bearing, running, or jumping.

Radiograph after 6 months of sirolimus treatment showed marked regression in the distal tibial osteolysis and complete resolution of the proximal tibial lesion (Figure 6d). Sirolimus dosage was thus reduced to 1 mg bd and continued for a further 6 months. At 12 months of sirolimus treatment, the distal tibial lesions had near-completely resolved with restoration of normal bony contour, re-establishment of cortical integrity, and substantial reduction in size of the talar lesion (Figure 6e). Sirolimus was continued at 1 mg bd for a further 1 year, and interval follow-up imaging showed further reduction in size of the tibial and talar lesions. At 25 months since starting sirolimus treatment, sirolimus dosage was gradually tapered, initially reduced to 0.7 mg bd for 1 month, then to 0.5 mg bd for 4 months, and then to 0.5 mg nocte for 12 months. Sirolimus was completely stopped after 3.5 years of treatment.

At 4 years since initiation of sirolimus treatment, the patient remains asymptomatic, enjoying full mobility, and did not encounter any adverse effects with normal full blood count, liver, and renal function tests throughout and after treatment. Patient remains in clinical remission at follow-up 6 months after termination of sirolimus treatment with no radiographic evidence of recurrence.

## Discussion

Currently, there is no uniform consensus on how to treat Gorham-Stout disease,^4^ with no recognised single treatment that has proven efficacy in halting disease progression. Gorham-Stout disease is characterised by a quite distinctive pattern of progressive bony dissolution^1^ which can lead to cortical breach and extension of lymphovascular proliferation into the soft tissues, mimicking a neoplastic process.^2^ It has a rather unpredictable course and prognosis as illustrated by the new osteolytic lesion emerging in the proximal tibia metaphysis at the nadir of the patient’s disease. Non-contiguous bone involvement is uncommon though recognised in Gorham-Stout disease.^5^ Conventional treatment includes surgery, radiotherapy, or pharmaceuticals such as bisphosphonates and alpha-2b interferon.^4^ Recently, targeting the vasculogenesis at the molecular level though inhibition of vascular endothelial growth factor (VEGF), by propanolol and sirolimus, have been tried, with 24 cases of sirolimus and 5 cases of propanolol treatment for Gorham-Stout disease showing variable outcomes to date,^6^ including 1 case of disease stabilisation with concurrent administration of both drugs.^7^ A recently published systematic review based on 73 articles, including 2 randomised-controlled studies, concluded that sirolimus may improve outcome of vascular anomalies though further data are required.^8^

Some studies have identified elevated angiogenic factors in Gorham-Stout disease. However, data on the use of sirolimus for treating Gorham-Stout disease are mainly from isolated cases, with 4 reported cases of disease stabilisation, 3 of poor response, and 17 cases of disease improvement, including 2 cases of recurrence after drug discontinuation.^6^ Recent clinical trials in USA and Japan report the efficacy of sirolimus for complex vascular anomalies, including Gorham-Stout disease, to be 50–67%.^9^ Similar to prior studies, sirolimus was well-tolerated by our patient who did not encounter any adverse effects throughout treatment. This case exemplifies the effective use of a stepwise drug regimen tailored to patient response, with gradual increase in sirolimus dosage upon initiation, followed by a static dosage during maintenance phase, and gradual tapering of dosage during withdrawal phase culminating in complete discontinuation of the drug.

The excellent response in our case strengthens the need for further research on the use of sirolimus for treating Gorham-Stout disease, possibly after an initial period of propranolol treatment. The unique treatment pathway of the presented case is that the patient was initially treated with propranolol and then sirolimus. Although this is only a single case, it is conceivable that propanolol may have downregulated angiogenesis within the lesion and thus primed the lesion for a favourable response to sirolimus. Such a serial treatment strategy has not been tried before.

At the cellular level, lymphovascular proliferation is driven by VEGF polypeptides, with VEGF-A principally involved in angiogenesis while VEGF-C and VEGF-D are principally involved in lymphangiogenesis.^10^ Propranolol is a beta-adrenergic blocker that acts solely on VEGF-A. Gorham-Stout disease may respond to propanolol.^10^ There has been a single report on disease stabilisation with concurrent propanolol and sirolimus dual-therapy.^7^ However, our patient had progressive osteolysis despite escalation of propranolol treatment. Sirolimus is a potent inhibitor of VEGF-C mediated mTOR phosphorylation that drives lymphatic proliferation.^9^ In our case, despite the predominance of angiomatous over lymphatic channels in the slide review of the biopsied sample (Figure 4), the profound response to sirolimus monotherapy shows how targeting lymphangiogenesis, even in the absence of lymphatic channels in the biopsy sample, can yield promising results. The dramatic response to sirolimus seems to refute the previous notion that Gorham-Stout disease is not dependent on lymphangiogenetic factor VEGF-C.^10^ This is in line with increasing scientific evidence that lymphatic proliferation plays a key role in Gorham-Stout disease.^4^

To our knowledge, this is the first report to show near-complete remission with sirolimus monotherapy following failure of propanolol treatment, adding strength to the evolving concept that targeting lymphangiogenesis can serve as an effective treatment direction for Gorham-Stout disease.

## Learning points

- Gorham-Stout disease is a rare, non-hereditary, progressive osteolytic disease due to uncontrolled proliferation of endothelial-lined vessels replacing normal bone, producing a characteristic pattern of bony dissolution with associated lymphovascular proliferation.
- In the presence of progressive disease despite escalation of propanolol treatment, a substantial response to subsequent sirolimus monotherapy was seen with near-complete remission, illustrating the role of lymphangiogenesis in the pathogenesis of Gorham-Stout disease and the potential benefit of serial propranolol followed by sirolimus therapy.
- Targeting lymphatic, rather than solely angiomatous, proliferation at the VEGF-level may be a promising future direction for the treatment of Gorham-Stout disease.
